# Corporate Culture Assessments in Integrative Oncology: A Qualitative Case Study of Two Integrative Oncology Centers

**DOI:** 10.1155/2013/316950

**Published:** 2013-05-30

**Authors:** Nadine Mittring, Marion Pérard, Claudia M. Witt

**Affiliations:** ^1^Institute for Social Medicine, Epidemiology and Health Economics, Charité-Universitätsmedizin Berlin, Luisenstraße 57, 10117 Berlin, Germany; ^2^Center for Integrative Medicine, University of Maryland School of Medicine, 520 W. Lombard Street, Baltimore, MD 21201, USA

## Abstract

The offer of “integrative oncology” is one option for clinics to provide safe and evidence-based complementary medicine treatments to cancer patients. As known from merger theories, corporate culture and integration models have a strong influence on the success of such integration. To identify relevant corporate culture aspects that might influence the success in two highly visible integrative oncology clinics, we interviewed physicians, nurses, practitioners, and managers. All interviews (11 in a German breast cancer clinic and 9 in an integrative medicine cancer service in the USA) were audio-recorded, transcribed and analyzed with content analysis. According to the theoretical framework of mergers, each clinic selected a different integration type (“best of both worlds” and “linking”). Nonetheless, each developed a similar corporate culture that has a strong focus on research and safe and evidence-based treatments, and fosters a holistic and patient-centered approach. Structured communication within the team and with other departments had high relevance. Research was highlighted as a way to open doors and to facilitate a more general acceptance within the hospital. Conventional physicians felt unburdened by the provision of integrative medicine service but also saw problems in the time required for scheduled treatments, which often resulted in long waiting lists.

## 1. Introduction

More and more people are suffering from cancer, and over 40% of adults suffering from cancer will use a form of complementary medicine (e.g., naturopathic treatments, acupuncture, etc.) during the treatment [1–3] with the aim of reducing side effects and enhancing their emotional and spiritual care [4]. An increasing number of oncology clinics are aware of this trend and are adapting to the patients' needs by providing integrative medicine services [5–7]. There has not yet been a clearly defined and established way to incorporate complementary medicine into conventional health care settings. A few theoretic models and frameworks for describing and evaluating integrative medicine have been published [8–10], and some integrative medicine centers have already been investigated [5, 11–14]. Integrative oncology is a growing field; it is mainly defined as the combined use of evidence-based complementary medicine with conventional medicine in cancer patients' care [15].

Integrative oncology may be viewed as a “merger” of two fields (conventional and complementary medicine) [16]. In business, a merger is the integration of two or more entities into one through a takeover or a pooling of interests. Corporate cultures of the entities have a very important influence on the success of the merger. The concept of corporate culture is best described by the phrase: “The way in which things get done within an organization” [17]. Two merging organizations must not necessarily have the same corporate culture, but they should be able to act together. The impact of the merger on the corporate cultures of both organizations is strongly influenced by the choice of integration type. According to Kummer [18], different degrees of integration are possible during a merger, ranging from a “confederation” type where both organizations work in parallel without any integration, to the integration type of the “best of both worlds” where a new organization is developed based on the advantages of both merging organizations.

Important aspects of corporate culture in a clinical setting are personal interests, management style, values, norms, communication culture, interaction with patients, and teamwork. When transferring this framework of corporate culture into medical systems, conventional medicine and complementary medicine could be viewed as two entities that pool their interests and form a new entity called “integrative oncology.”

The field of integrative oncology is growing, and developing recommendations as to how its implementation will work best to support the needs of patients and professionals are critical. As a first step, it is helpful to have a closer look at the corporate culture of the integrative oncology centers that already exist [19]. We conducted two case studies in order to evaluate aspects of corporate culture in highly visible integrative oncology centers at hospitals.

As the clinic structures in countries differ, we decided to focus on two different clinics, one in Germany and one in the USA. In the case studies, we focused on the elements of corporate culture mentioned above as well as conducted additional inquiries regarding: type of cooperation between complementary and conventional medicine (= integration model), therapeutic services and offerings, advantages and disadvantages of the cooperation, aspects of communication and teamwork, role of evidence, research and safety, and resources and strategy.

## 2. Methods 

### 2.1. Study Design

The study was a semistructured qualitative interview study [20] consisting of two case studies conducted in 2012. Two of the authors (C. M. Witt and N. Mittring) are trained in qualitative research methods, and two of the authors have business backgrounds with knowledge in merger theories (M. Pèrard and C. M. Witt).

### 2.2. Study Sample

Clinics were chosen due to their visibility. In Germany, the first center asked confirmed participation. In the USA, we asked the two largest cancer centers; one declined participation in our study; the other confirmed participation. The recruitment occurred via personal contact by the PI of the study, who invited the directors of the clinics to participate. The study was approved by the Institutional Review Board of the Charité Universitätsmedizin Berlin, Germany (EA1/293/11), and by both clinic administrations.

The aim was to gather different perspectives by interviewing staff with different competencies and opinions that played a role in the integrative medicine structures within the clinic.

The interview guidelines for the first case study were developed by the authors based on the literature on corporate culture aspects in mergers [17, 21–23] and integrative oncology. There were different interview guidelines for each target group (administration, medical doctors, nurses, and patients).

The first case study took place in a breast cancer clinic in Essen, Germany, in January 2012. Two of the authors (C. M. Witt and M. Pèrard) conducted all interviews together over a 2-day period. Furthermore, they collected leaflets and took notes of observed processes in the clinic. The interview guidelines for the second case study were revised based on the preliminary results of the first case study and contained further questions concerning aspects of corporate culture.

The second case study took place in Houston, USA in July 2012, and built on the results of the first case study. Two of the authors (C. M. Witt and N. Mittring) conducted all interviews together during a 2-day stay in Houston, collected leaflets, and took notes. 

Interviews were conducted face-to-face. Written informed consent was provided by all of the interviewees. All interviews were digitally recorded, and a short interview protocol of every interview was written.

### 2.3. Data Analyses

The interviews of each case study were transcribed verbatim. Analyses followed a content analysis approach according to Mayring [24] assisted by the software MAXQDA [25]. Coding took place in several rounds. First, the themes of the interview guidelines were used to organize the materials and provided the initial codes. Then, each segment was analyzed according to the themes present. The results of the analysis of the first case study served as material to revise the questions for the second case study. The analysis process of the second case study used the analysis process of the first case study. Finally, categories that arose during analysis were bundled into core categories, and all analysis results were brought together and compared. Data from the notes and the leaflets of the clinics completed the results. Written memos during the analyses supported the analyses and results. Analyses and results were regularly discussed in the research team and in a qualitative research group to ensure reliability, validity and grounding of results in the material.

## 3. Results

### 3.1. Sample

The sample consisted of eleven interview participants in Germany and nine in the USA (see Table 1).

#### 3.1.1. Short Description of the Two Centers

*Integrative Oncology for Breast Cancer Program, Essen*. The Integrative Oncology for breast cancer program was developed as a model in cooperation with the *Department for Senology* and the *Department for Complementary and Integrative Medicine* (Chair for Complementary Medicine University Duisburg-Essen) and is part of the Kliniken Essen-Mitte, Germany, an academic teaching hospital of the University of Duisburg-Essen. It originated in the year 2010 and is a highly specialized clinic for breast cancer patients where conventional and integrative medicine staff works together in one team and one department. The Department for Complementary and Integrative Medicine in the clinic (since 1999) includes a day clinic for oncology patients and inpatient treatment of chronic diseases. The integrative medicine treatments are provided by physicians specialized in complementary medicine and by trained therapists (e.g., therapists specialized in mind-body medicine) [7]. The concept of the model used in Essen is based on the integrative medicine model of Memorial Sloan-Kettering Cancer Center in New York, and some staff members were trained there.

*MD Anderson Integrative Medicine Program, Houston*. The University of Texas MD Anderson Cancer Center is located in Houston, Texas, USA, on the campus of the Texas Medical Center. MD Anderson is the largest cancer center in the USA for cancer patient care, prevention, research, and education [5]. The integrative medicine program has a focus on clinical care, research, and education and is part of MD Anderson and located in the same buildings. The integrative medicine center (the clinical delivery center of the program) started in 1998 as the “Place of Wellness” and changed its name in 2007 to “The Integrative Medicine Center.” It is a referral service with its own team open to all conventional departments and includes inpatient and outpatient services. A consultation service that offers information about complementary medicine is provided by physicians specialized in both oncology and complementary medicine, and treatment is provided by trained therapists (e.g., acupuncturists). 

### 3.2. Results of the Case Studies

#### 3.2.1. Integrative Oncology for Breast Cancer Program, Essen

*Integration Model.* Based on merger theories, the model used for integration can be described by combining the “best of both worlds.” In Essen, the best of both entities—the *Department for Senology* and the *Department for Complementary and Integrative Medicine*—was merged into a new program called “integrative oncology for breast cancer patients.” Because the clinic was newly developed, the superior elements of both conventional and complementary medicine could be identified and integrated into the new program to offer the best possible care. Furthermore, both partners could maintain their advantages [7, 18].

*Philosophy and Services.* The Integrative Oncology for breast cancer program set great value on a holistic and patient-centered approach in the interaction with the patient. The high degree of specialization in breast cancer increased the quality of patient care and allowed optimized processes in the team. The integrative medicine offerings were provided in addition to the conventional cancer treatment and include treatment options such as acupuncture, massage therapy, naturopathic treatments, and mind-body medicine (e.g., Qigong). Main indications for these treatments were side effects of the conventional cancer treatment and supporting the patients in coping with cancer. The treatment focus is on nonpharmaceutical interventions. Several times, the importance of individuals for the success of the project was highlighted. These individuals, who unified positive personality aspects and medical competencies, were highly important for positive development of the center. Key aspects of success were identified as individuals' great motivation to push the project forward, and the ability of these individuals to motivate other team members.

*(…) There's a need for the human element (…). Both the patients and the colleagues take [this person] seriously; it's important to cultivate a relationship that's characterized by cordiality and empathy. The worst thing that could happen is to feel not taken seriously, or to feel bothersome. I think this relationship between these key individuals and others is quite important in such an innovative project.”—Chief physician specialized in integrative medicine.*

The working atmosphere in the Integrative Oncology for breast cancer program was found to be very good and relaxed by most interview partners, although their work schedule was busy. Self-care of the employees played an important role in the center. Several offerings (e.g., weekly team yoga) were well received by the staff.

*Professional Team.* Interview participants saw regular and detailed team meetings as very important and essential for the successful implementation of integrative medicine and a good team structure. A weekly meeting with all members of the team was established in spite of implementation problems in the beginning. The meetings were used to discuss and inform all members about patients and their individual treatment concepts and the harmonization of conventional and complementary medicine treatments and to solve emerging conflicts within the team.

*“We have a lot of conversations here. We have meetings, every Monday, for example, there's a big meeting where everybody joins in. Everybody has the opportunity to say something, (…) we have a lot of dialogue and sharing.”—Conventional nurse.*

The multidisciplinary discussion of the patient cases during those meetings relieved patients of the necessity to endlessly repeat their stories to each clinician involved in their treatment. The integrative medicine team and the conventional physicians sought to combine rounds in the beginning, but this was not practicable due to different round styles and timelines. 

*Interaction with Patients*. The integrative medicine physicians and therapists in the Integrative Oncology for breast cancer program dedicated a lot of time to talking and listening to the patients. This facilitated an intense and open relationship with the patients and a better understanding of each patient's individual needs and wishes. Furthermore, it unburdened the conventional physicians because they were able to refer patients with concerns and questions to their integrative medicine colleagues. Thus, the team was able to treat a higher number of patients with the same quality of patient-care.

*“When I'm sitting in the consultation [naturopathy], I have the feeling that the clocks stop. Then it seems I have all the time in the world just for myself (…). The things that I did not have enough time to ask about or explain, [in the consultation with the conventional physician], or the things I've forgotten, I can always talk about with the complementary medicine physician.”—Patient.*

That the treatments are based on positive evidence was emphasized by the patient and led to more trust in the treatments.

*Resources*. The Integrative Oncology for breast cancer program experienced a high demand among patients with increasing case numbers over time. Therefore, the time and space resources for integrative medicine treatments were quite tight, as the treatments requested required special rooms and were more time-consuming. The consequence was an increase in waiting periods for the patients to receive treatments, leading to disappointment among some of the integrative medicine therapists.

Some of the integrative medicine practitioners mentioned that due to the short length of stay in a surgical department such as the breast cancer center, it was not really possible to offer the patients a holistic naturopathic treatment. Therefore, patients were offered the opportunity to continue treatment at the integrative oncology outpatient clinic, located in the same hospital. 

*Visibility.* The Integrative Oncology for breast cancer program had very high visibility in German-speaking countries and included broad media attention and good visibility on an international level. From the beginning, the integrative oncology project was fully supported by the CEO who contributed to the project's visibility and acceptance across the whole clinic. Such support from high-level management opened many doors for integration. As other department heads observed how successfully the program worked, all other oncologists in the hospital also demonstrated an interest in integrative oncology.

*Research.* Research and evidence-based medicine (EBM) played an important role for the breast cancer clinic. The breast cancer center had implemented an innovative and high-level, evidence-based information database called “SenoExpert” that offers an individual analysis of the current evidence concerning the therapeutic options for an individual patient [7, 26]. Integrative medicine offerings were subsequently included in the SenoExpert database.

#### 3.2.2. Integrative Medicine Program, Houston

*Integration Model. *Based on merger theory, the type of integration that characterizes the Houston project can be described as “linking,” which means that two medical approaches are linked with each other, here in the form of a referral service [18]. The integrative medicine program did not operate independently and autonomously before the project began but rather was developed for the purpose of integration to meet patient needs. The program offers services to a variety of departments and maintains a high level of autonomy and an independent culture.

*Philosophy and Services*. The integrative medicine team in Houston placed a high value on research, safety, and evidence-based medicine. It was very important for them to treat the patients according to the principles and philosophy of the larger cancer center. They treated the patients strictly following evidence-based medicine using integrative medicine in support of the conventional oncology treatment. An effort was made to communicate this important aspect with the primary oncology team. Patients who strongly demanded treatments that were not seen as evidence-based were not able to receive these treatments in the clinic. The use of supplements during conventional oncology treatment (e.g., vitamins, minerals, and herbs) was discouraged unless supported by clinical research. The importance of safety and positive evidence for a treatment was also communicated to the patients during the consultation regarding complementary medicine. Furthermore, there was a clear emphasis on the term “integrative medicine” instead of employing “conventional and complementary medicine.” As the Medical Director of the service was trained in both oncology and complementary medicine modalities, he automatically incorporated his conventional training into his work at the integrative medicine center. 

Holistic treatment of the patient and a patient-centered approach were core aspects of the service with a focus on the quality of life and an improvement of the patient's outcomes. The integrative medicine center provided individual services (consultation service on complementary medicine, nutrition, and physical activity, as well as treatments with acupuncture, massage therapy, music therapy, and meditation). Furthermore, the center offered group programs (e.g., music therapy, meditation, Tai Chi, and cooking classes). Main indications for the use of the service were side effects, for example, of chemotherapy (hot flashes, nausea, etc.) and support for coping with cancer. Most treatments and services were primarily provided for patients, but massage and group programs were also offered to family members and caregivers of the patients.

*Professional Team and Communication.* The whole integrative medicine team held weekly multidisciplinary meetings to discuss more difficult cases and patients' treatments and to solicit feedback from everyone in the team. Referring colleagues from the conventional side did not take part in the team meetings. Once a month, a team meeting was held with physicians from other supportive care centers to review shared patient cases (psychiatry, palliative care and rehabilitation medicine, fatigue center, etc.). The communication between the integrative medicine team and the referring departments was driven mainly through patient records and e-mails. It was important for the integrative medicine center team to give the referring physicians personal feedback about the experiences in the consultation with the patient and recommended treatments. The referral and feedback system was established between the integrative medicine center physicians and the referring oncologists. The cancer nurses had full access to patients' records, but not to the e-mails, and suggested that they also would be interested in receiving more direct and detailed information. 

The Medical Director of the integrative medicine center acted as a door opener for the project as he was able to cultivate a good relationship with the conventional oncologists; he spoke their language and had the same knowledge base. He could thereby effectively communicate with the conventional physicians, garner their trust, and educate them about the integrative medicine model.

*“[A staff member asked me:] “What do you consider yourself; who are you? What would you say you are- an oncologist, palliative doctor, or an integrative medicine doctor?” And I said, “Well, I'm an oncologist first.” And they said, “That's the right answer.” Because at MD Anderson, it's a cancer center. You have to come in the door as an oncologist. And then later on, you can say, “Oh, I do palliative, I do integrative.” But you knock on the door as an integrative medicine doctor, they go, they do not want to open the door, right? They do not know who you are. So I think that made a big difference.”—Medical Director, Integrative Medicine Center.*

Overall, the interview participants from the integrative medicine cancer service reported a motivating and positive working atmosphere with importance placed on the self-care of the employee, (e.g., they offer a weekly meditation class).

*Interaction with Patients.* Shared decision making and respect for patients' choices and wishes were very important aspects of patient interaction. The integrative medicine concept included time for talking and listening to the patient. An hour was regularly scheduled for the first consultation with a physician in the integrative medicine center. This allowed an intense and open relationship with the patient and the opportunity to get deeper insight into the patients' needs and perceptions about their diseases and treatment decisions. This adjustment was seen as a big relief for the conventional departments as the patients were intensively cared for and had someone to talk to about their questions regarding all therapeutic options, including conventional treatments when they arose.

*Resources*. The demand of the oncology departments for the integrative medicine center was higher than the available resources. Therefore, long waiting lists became standard for integrative medicine services. Conventional oncologists carefully chose patients who required an integrative treatment, as they wanted to use their scarce resources as effectively as possible.

*“I think the biggest issues we have, is it's, I do not think it [the integrative medicine center] is resourced adequately for the size of the institution. I think I personally would be referring more patients if they had more physicians and more time to, to manage the patients. I try to limit my use of the integrative medicine center.”—Conventional oncologist.*

Due to limited resources, the integrative medicine team promoted its service only in a passive way, which means that they did not promote it actively in every oncology department but only in the departments that asked them to present their concept and the services they offer.

Another problem was that the integrative medicine center did not bring much added revenue to MD Anderson. Therefore, MD Anderson administration had reduced motivation to invest in and increase the service in spite of the high need and demand.

*Visibility*. The integrative medicine center was known throughout the hospital because of its regular and public presentations on the evidence of the offered services.

The education of the conventional departments about integrative medicine was not officially integrated in, for example, the physicians' education program, with the exception of the monthly grand rounds, but the conventional oncologist who mainly referred to the service tried to create awareness and open-mindedness towards the integrative service within the team. 

*Research*. Research played a key role for the integrative medicine program as a whole. The participants described experiencing more respect and acceptance across the hospital because of their well-funded research program and cooperative projects with other departments. The research and grants of the director of the department were highly respected in the hospital and were seen as another door opener.

*The other thing that I think is, (…) what has given us tremendous recognition and acknowledgement in the institution of being an area that needs to be supported is the research portfolio. So, and in particular in the division of cancer medicine, a large portion of the research funds are coming from industry and it's not that common for faculty to have NIH-funding. Now we have a lot of NIH-money and (…) when they [the conventional departments] see that an acupuncture grant gets a perfect score at the NIH, when they see (…) that we're not just doing this really merely at random but we're really studying it, and following the biomedical model.—Director, integrative medicine program.*

It was very important for the interview participants to conduct studies with the treatment modalities they offered to the patients to contribute to the available evidence. They also had a monthly research meeting where research ideas were discussed, and trials were developed and monitored with the goal of fostering more research.

## 4. Discussion 

There is an increasing demand for integrative oncology, and therefore integration processes in this field need to be analyzed and described. We investigated aspects of corporate culture in two highly visible integrative oncology clinics.

Although differing in their type of integration model, the two centers showed a lot of similarities regarding their philosophies and priorities. Both clinics had a great focus on research and evidence with the goal to offer evidence-based treatments. Research was an important pillar for the projects to achieve more visibility and acceptance within the whole hospital. This demonstrates the general trend in the academic world towards evidence-based medicine [27, 28] and the possibility for scientifically based integrative medicine treatments to be established in the clinics [29]. 

As both integrative oncology clinics experienced very high demand and dedicated a lot of time to patients, the two had to manage tight time and staff resources. On the other hand, the time-consuming patient care led to positive and intense patient contact and reduced the burden on the conventional oncologists and nurses by satisfying patients' needs to talk and to be heard and informed about complementary medicine. Both integrative clinics also placed a great value on a holistic and patient-centered, individualized approach, which is one of the hallmarks of integrative medicine [10, 30, 31]. 

Both integrative medicine centers had at least one conventional physician as part of the team who acted as a door opener for the project regarding other departments and the public. This was a key aspect for the visibility and acceptance of the project. The importance of particular persons for integrative medicine projects has already been highlighted in other studies [30, 32]. 

The impact of the merger of conventional and complementary medicine on the corporate culture is strongly influenced by the integration type, which is used within a merger. 

According to the theoretical framework of mergers, both explored centers have chosen quite different integration types [18] and could not be compared point by point, while the type of merger integration in the newly established German breast cancer center can be most accurately characterized as the “best of both worlds” with a high level of structural and spatial integration within one entity. Thus, it generated a need for a strong new corporate culture that represents a mixture of both partners. The integrative medicine center in the USA was implemented in an already existing clinic model and used the “linking” integration model. Although in the clinic in the USA there is an overlap of the cultures of the conventional departments and the integrative service, the conventional departments and the integrative service are still independent entities with their own cultural characteristics. Other theoretical frameworks could be applied to the case studies. Regarding the theoretical framework of integrative medicine, which was described by Boon and colleagues [8] and included seven models of team-oriented health care practice, the USA integrative medicine cancer service can be classified as a “multidisciplinary” model with two individual teams (the conventional and integrative medicine professionals work in separate teams), while the breast cancer clinic provides an example of the “integrative” model with a multidisciplinary team (conventional and integrative medicine professionals) that practices consensus building.

Like all research projects, this study contains limitations. We investigated and compared only two integrative medicine centers, both offering services to inpatients and outpatients. Our results are based on the interviews and the provided materials. Therefore, the information that the clinics had a strong focus on evidence-based medicine is based on the abovementioned methods and not on critical appraisal of the literature. The field of integrative oncology in Germany and the USA is heterogeneous ranging from private practitioner practices to services offered in established cancer centers. Our case studies focused on the established cancer centers. Therefore, the aspects discussed here have less relevance for private practices. It is possible that in other settings, for example, centers that offer only outpatient care, different key themes, and cultural aspects that we did not capture with our study would play a role. However, the high number of similarities within the important aspects of communication, professional team, and philosophy, despite the large difference of context of the two clinics in two countries, suggests that studies of other similar integrative medicine settings will not bring many new key aspects. Though we suppose that the investigated centers have successfully incorporated a model of integrative oncology, we cannot be sure that the identified characteristics in fact aspects leading to successful integration in other clinics. We only interviewed one patient in the German case study, and we did not get the patient's perspective from the integrative medicine center in the USA. The patient perspective is underrepresented and could have provided interesting insights for comparison. The research question of how to determine aspects of corporate culture in integrative medicine centers required a qualitative study design that incorporated the possibility of acquiring an indepth insight into the setting [20]. Adding focus groups and participant observation to the applied methods might have brought different aspects and provided a broader picture. However, the results might have been biased by the selection of interview partners and their effort to “put the best face” on their center. 

These case studies identified some aspects which may support integration when developing new projects in integrative oncology. The integration type has a strong impact on the corporate culture. Because of this, first, the adequate model of integration of complementary and conventional oncology must be defined before implementation and depends strongly on the clinic's own resources and needs. The chosen model should be necessarily adapted to clinic factors such as the type of department using the integrative medicine service, the average length of patient stay, and possible ways of reimbursement for integrative medicine treatments. Furthermore, it is advisable to adapt the integrative medicine offerings to the needs and processes of the oncology departments. This includes aspects such as time and space needed for the treatments.

Another very important aspect is to clarify the roles of the different partners and treatment modalities within the integration model from the beginning and make them transparent to everybody in the team through open communication [5, 19]. Either a good feedback system or regular meetings of the partners involved in the patients' treatments are very advisable to stay in touch, resolve conflicts, and ensure an intense exchange about the patients in common.

Overall, visibility of the integrative oncology project is a key element and can be achieved by representing the project on a constant basis in the clinic.

A conventional physician (door opener) working in the integrative oncology service, or at least supporting it, can be very helpful to improve the standing within the clinic and promote the project among other conventional colleagues [12, 32]. Conducting research projects on integrative medicine and cooperation studies with other departments can act as a door opener to conventional departments within the clinic and improve the acceptance of integrative oncology.

And most importantly, having positive evidence on effectiveness and safety for the offered treatments is key in fostering trust among patients and other departments. 

Considering all these aspects can make integrative oncology as a treatment concept highly adaptable to the needs of the individual patient and successfully established in a broad manner in the field of oncology.

## 5. Conclusion

Despite using different integration models, both integrative oncology clinics have developed a similar corporate culture that has a strong focus on research and evidence-based treatment and fosters a holistic and patient-centered approach.

## Figures and Tables

**Table 1:** Description of interview participants.

	Breast cancer clinic, Germany	Integrative medicine cancer service, USA
Leading administrative person	1	1
Conventional oncologist	2	1
Physician specialized in complementary medicine	2	1
Psychologist specialized in complementary medicine	—	1
Conventional nurse	2	2
Nurse specialized in complementary medicine	—	1
Therapist specialized in complementary medicine	3	2
Patient	1	—

Total	11	9
